# PD177 The Effect Of COVID-19 On Cancer Screening In Brazil, Canada, And The USA: A Cross-National Study

**DOI:** 10.1017/S0266462324004021

**Published:** 2025-01-07

**Authors:** Guvenc Kockaya, Yaren Erkut, Selin Okcun, Ekin Begum Ozdemir, Mustafa Kurnaz, Birol Tibet

## Abstract

**Introduction:**

The COVID-19 pandemic strained hospital systems and diverted resources, prompting a reorientation of healthcare priorities. This shift disrupted patient access to preventive cancer screenings and curtailed interactions between medical professionals and patients. This study aimed to examine changes in cancer screening during the COVID-19 pandemic period (2019 to 2021) in Brazil, Canada, and the USA.

**Methods:**

The study included a literature review of academic articles, health reports, and government data that focused on the impact of the pandemic on cancer screening. Official health data in Brazil, Canada, and the USA were collected from medical records, national health databases, and screening statistics. A comparative analysis was conducted to unveil the changes in access to screening services for colorectal cancer (CRC), breast cancer, hepatocellular carcinoma (HCC), and cervical cancer.

**Results:**

During the COVID-19 period, significant declines in cancer screening were observed globally. In Canada, CRC diagnoses dropped by 55 percent and remained 20 percent lower than averages from previous years, with an estimated 467 cases undiagnosed by August 2020. In the USA, HCC screenings were reduced by 44 percent, while cervical cancer screenings for women aged 21 to 29 years plummeted by 78 percent. Additionally, mammography screenings fell drastically from 180,724 in March to May 2019 to just 1,681 in the same period of 2020, leading to fewer breast cancers detected and a surge in symptomatic, aggressive tumors. Similarly, Brazil saw a 39 percent drop in breast cancer screenings.

**Conclusions:**

The COVID-19 pandemic significantly disrupted cancer screening programs across Brazil, Canada, and the USA, resulting in marked declines in the number of diagnoses of various cancers. This reduction highlights the extensive impact of the pandemic on preventive health care, necessitating strategies to address the backlog and ensure timely cancer detection and treatment in the post-pandemic era.

